# Monkeypox in-patients with severe anal pain

**DOI:** 10.1007/s15010-022-01896-7

**Published:** 2022-08-12

**Authors:** Frieder Pfäfflin, Daniel Wendisch, Roland Scherer, Linda Jürgens, Gisèle Godzick-Njomgang, Eva Tranter, Pinkus Tober-Lau, Miriam Songa Stegemann, Victor Max Corman, Florian Kurth, Dirk Schürmann

**Affiliations:** 1 Department of Infectious Diseases and Respiratory Medicine, Charité - Universitätsmedizin Berlin, Freie Universität Berlin and Humboldt-Universität Zu Berlin, Berlin, Germany; 2 Center for Intestinal Surgery and Coloproctology, Krankenhaus Waldfriede, Berlin, Germany; 3 Department of Hematology, Oncology, and Tumor Immunology, Charité-Universitätsmedizin Berlin, Freie Universität Berlin and Humboldt-Universität Zu Berlin, Berlin, Germany; 4 Institute of Virology, Charité - Universitätsmedizin Berlin, Freie Universität Berlin, Humboldt-Universität Zu Berlin, Berlin, Germany

**Keywords:** Monkeypox, MPX, MPXV, Germany, MSM, HIV

## Abstract

Berlin is amongst the cities most affected by the current monkeypox outbreak. Here, we report clinical characteristics of the first patients with confirmed monkeypox admitted to our center. We analyzed anamnestic, clinical, and laboratory data. Within a period of 2 weeks, six patients were hospitalized in our unit. All were MSM and had practiced condomless receptive anal intercourse in the weeks preceding admission. The chief complaint in all patients but one was severe anal pain unprecedented in severity. Investigations revealed proctitis, as well as anal and rectal ulcers with detection of monkeypox virus. Our findings support the hypothesis that sexual transmission plays a role in the current outbreak.

## Introduction

In May 2022, the United Kingdom Health Security Agency reported patients with monkeypox (MPX) in the UK, followed by increasing case numbers in several cities in Europe and the U.S. [1, 2]. Berlin is amongst the cities most affected by the current global MPX outbreak with 617 of a total of 874 cases reported in Germany as of June 29, 2022 [3, 4]. Here, we report clinical characteristics of the first six patients with confirmed MPX admitted to the Department of Infectious Diseases of Charité—Universitätsmedizin Berlin, Germany, the regional referral center for patients with MPX needing hospitalization.

## Methods

The patients described in this case series are the first six patients included in an ongoing prospective clinical cohort study, with the aim to analyze clinical and laboratory characteristics of MPX. Patients were included from May 27 to June 10, 2022, after written informed consent had been obtained. Treatment of patients, clinical sampling, investigations and documentation were carried out according to clinical necessity. The study is approved by the ethics committee of Charité—Universitätsmedizin Berlin (EA2/139/22) and is performed in accordance with the ethical standards laid down in the 1964 Declaration of Helsinki and its later amendments. All patients provided written informed consent to publication of their data and one patient additionally to publication of a photograph.

## Results

**Fig. 1:**
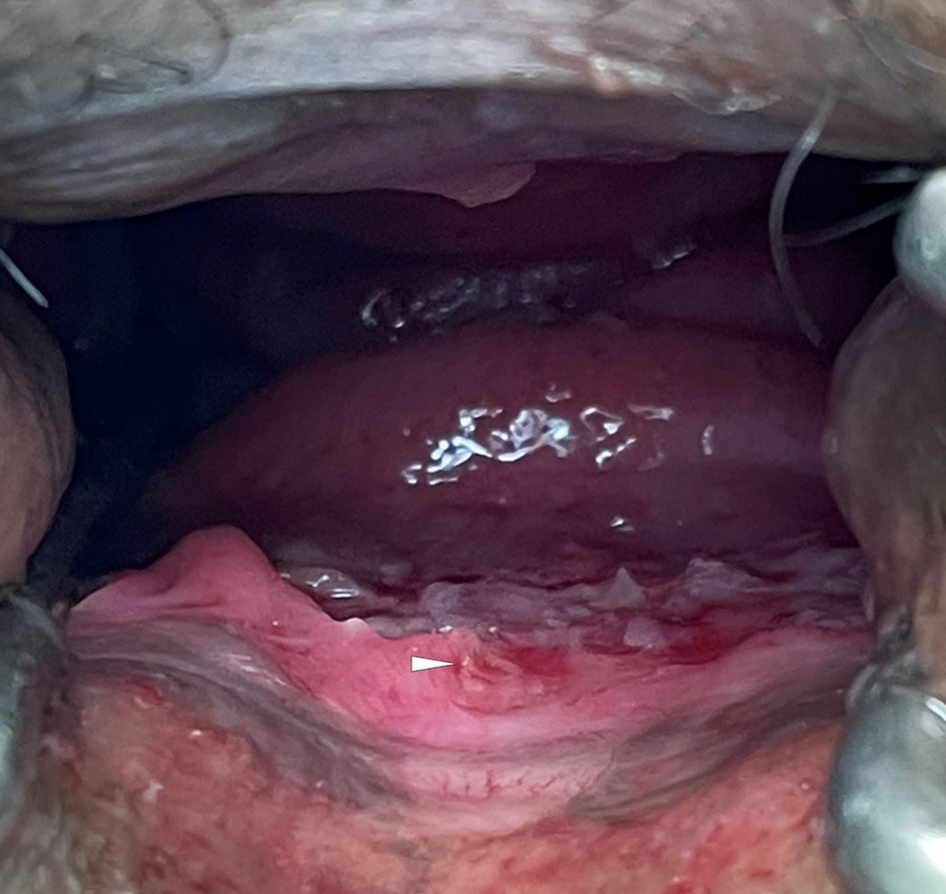
Proctoscopy under spinal anesthesia in lithotomy position reveals severe proctitis with ulceration at 5–7 o’clock. A small monkeypox lesion can be seen at 6 o’clock in the anoderm (white arrow)

From May 27 to June 10, 2022, six patients with non-travel related monkeypox detected by PCR in fluid from cutaneous blisters were hospitalized in our unit (GenBank: ON813251.2). Details of the clinical courses are summarized in the Table 1. All patients self-identified as men who have sex with men (MSM) and had practiced condomless receptive anal sexual intercourse with different partners in the weeks preceding admission. None of the patients was immunocompromised. Two patients were living with HIV and on anti-retroviral therapy (ART) with well controlled infection, three patients were on pre-exposure prophylaxis (PrEP). Prodromal symptoms were mild and had subsided spontaneously before admission. The number of concurrent lesions ranged from three on one arm to a maximum of 36 with a wide distribution over the body surface. Concurrent STDs were detected in three patients: two patients with gonorrhea, both detected with PCR (COBAS^®^, Roche, Basel, Switzerland), and one patient with gonorrhea, *Ureaplasma* and *Mycoplasma hominis,* each detected with PCR (COBAS^®^ for gonococci and RIDA^®^ GENE STI, R-Biopharm, Darmstadt, Germany for Ureaplasma and Mycoplasma hominis). The general condition was good in all but one patient who had extended inflammation of the sigmoid, rectum, and anus. One patient suffered from a perianal abscess, which was treated by incision and drainage. The abscess had appeared prior to the onset of prodromal symptoms and the other skin lesions and was thus considered not to be related to monkeypox. The chief complaint of all other patients was intense anal pain. These patients rated the pain as 9–10/10 on the numerical rating scale (NRS, with zero representing 'no pain at all' and ten representing 'the worst pain possible’). The character of the pain was mostly described as unprecedented in severity (although all patients had experienced anal disorders in the past), stabbing, burning, and unbearable on defecation. Three of these patients underwent proctoscopy under spinal anesthesia to rule out anal abscess. Proctoscopic findings were severe proctitis, anal and rectal ulcers (Fig. 1). Computer tomography (CT) of the pelvis was performed in one patient. CT scans showed severe inflammation of the sigmoid, rectum, and anus. Anal lesions were positive for monkeypox DNA in all three cases where swabs were obtained. All patients were treated with systemic and local analgesics (some doses of the systemic analgesics were administered intravenously). Stool softeners were given to ease the aggravated pain during defecation. The intense pain lessened within a couple of days in all patients. The duration of hospital stay ranged from 3 to 6 days. All symptoms from all patients had much alleviated at hospital discharge.

**Table 1:** Clinical and laboratory characteristics of six patients with monkeypox

	Patient 1	Patient 2	Patient 3	Patient 4	Patient 5	Patient 6
Sex	Male	Male	Male	Male	Male	Male
Age range	41–50	21–30	31–40	31–40	21–30	31–40
Mode of infection (sexual preference)	MSM (insertive anal, receptive anal)	MSM (insertive anal, receptive anal, oral)	MSM (receptive anal)	MSM (receptive anal)	MSM (receptive anal)	MSM (receptive anal)
Prodrome	Fever	Fever, malaise	None	Fatigue	Fever, malaise, myalgia, sweats	Myalgia, fever, malaise
Lymphadenopathy	Left inguinal	None	None	Left inguinal	None	none
Number of concurrent lesions	15	5	7	≈ 20	36	3
Distribution of lesions	Limbs	Left arm	Limbs	Arms, trunk, genital	Head, neck, trunk, limbs	Legs
HIV status	Positive	Negative	Negative	Negative	Negative	Positive
CD4 count (if HIV positive)	870/µl					> 500/µl
Concurrent STD (site of diagnostic sample)	None	None	None	Syphilis (blood), gonorrhea (rectal)	Gonorrhea, Ureaplasma, Mycoplasma hominis (all urethral)	Gonorrhea (rectal)
Treatment of concurrent STD				Penicillin G benzathine, ceftriaxone	Ceftriaxone, azithromycin	Cefriaxone, azithromycin
Chief clinical complaint	Perianal pain	Anal pain	Anal pain	Anal pain	Anal pain	Anal pain
Maximum severity of anal pain	5/10	10/10	9/10	10/10	10/10	10/10
Character of anal pain	Stabbing, pressing	Unprecedented in severity, stabbing, burning, aggravation on defecation	Unprecedented in severity, pressing, stabbing, burning, aggravation on defecation	Aggravation on defecation	Unprecedented in severity, burning, stabbing, aggravation on defecation	Burning
Anal investigation for specific causes			Proctoscopy under spinal anesthesia	Proctoscopy under spinal anesthesia	CT scan of pelvis	Proctoscopy under spinal anesthesia
Finding on ano-rectal investigation	Anal abscess	Anal fissure	Rectal ulcer, proctitis	Anal ulcer	Inflammation of sigmoid, rectum and anal canal	Anal ulcer, proctitis
Other findings	Histology from anal abscess: extensive suppurative, partially hemorrhagic- necrotizing inflammation of corium and subcutis		Histology from ulcer: rectal ulcer with crypt hyperplasia and interstitial stromal fibrosis			
Specific management of complications	Incision and drainage					
Analgesia (per os if not indicated differently)	Ibuprofen 600 mg q8h	Metamizole 500 mg q6h, tramadol 50 mg q12h, lidocaine 50 mg/g topical	Ibuprofen 600 mg q8h, metamizole 500 mg q6h intravenously, lidocaine 50 mg/g topical	Metamizole 500 mg q6h, lidocaine 50 mg/g topical	Metamizole 500 mg q6h intravenously, lidocaine 50 mg/g topical	Metamizole 500 mg q6h, lidocaine 50 mg/g topical
Other supportive care	Macrogol, rinsing with polyhexanide	Macrogol	Macrogol	Macrogol	Macrogol	Macrogol
Duration of clinical course prior to admission	14 d	9 d	11 d	5 d	2 d	5 d
Duration of hospital stay	4 d	3 d	3 d	3 d	6 d	3d
Outcome at hospital discharge	Improved	Improved	Improved	Improved	Improved	Improved

MSM, men who have sex with men; STD, sexually transmitted disease; CT, computer tomography

## Discussion

We report on six monkeypox cases hospitalized in a single center. Notable seems the homogeneous clinical presentation with intense anal pain in all but one patients, which was described as unprecedented in severity, despite a history of anal disorders in the past. The pain is in contrast to findings from recent studies from Nigeria, the Democratic Republic of the Congo (DRC) and the UK prior to the current outbreak, where anal pain was not reported [5–7]. All patients had practiced condomless receptive anal intercourse in a time window compatible with time of infection. Seminal fluid has been shown to be positive for MPX DNA earlier [8] and MPX DNA was also detected in one sample we tested. Anal lesions were positive for MPX DNA in all three of our cases where swabs had been obtained, a finding also reflected in other reports [8]. Our findings therefore strengthen the hypothesis and current perception that sexual transmission plays a role in the current outbreak. In a recent study from the Democratic Republic of the Congo, the most common exposures to monkeypox were cleaning/dressing/consumption of wild game and handling uncooked, freshly butchered meat. The most common clinical complaints were rash, malaise, sore throat, lymphadenopathy (mostly cervical), and anorexia [7]. Thus, in the study from the DRC, exposure of skin and pharynx led to affection of the skin and the gastrointestinal tract. Contrary to that, our patients suffered from anal pain after receptive anal intercourse. We therefore hypothesize, that the route of transmission conditions the clinical course. The clinical presentation observed in our patients merely reflects the extreme end of anal involvement in monkeypox. Of note, positive anorectal MPX PCR results from asymptomatic persons have been described recently [9].

None of our patients was treated with tecovirimat. The availability of tecovirimat in Germany was limited to five treatment courses for the whole country. Requests for tecovirimat had to be discussed within the working group of competence and treatment centers for high-consequence infectious diseases (Ständiger Arbeitskreis der Kompetenz- und Behandlungszentren für Krankheiten durch hochpathogene Erreger (STAKOB) at the Robert Koch-Institute (RKI)). We did not request tecovirimat for our patients as none of them was immunocompromised and no immediate danger for critical organs was perceived. However, we do not know whether administration of tecovirimat would have alleviated the intense pain. We did neither request brincidofovir as this drug was not available in Germany and treatment can be associated with side effects, hepatotoxicity in particular [6]. The World Health Organization (WHO), in its interim rapid response guidance, recommends antivirals to be preferentially administered within randomized clinical trials [10].

From May 27, to June 10, 2022, six out of 120 patients with confirmed monkeypox in Berlin were hospitalized in our center. Further six of 497 patients were treated as in-patients as of June 29, 2022. The decreasing hospitalization rate may reflect growing competence with the management of monkeypox cases. Of note, no cases with critical or life-threatening clinical conditions have been reported so far. Although severe, the pain described by our patients responded to analgesics.
